# Next-generation sequencing using a pre-designed gene panel for the molecular diagnosis of congenital disorders in pediatric patients

**DOI:** 10.1186/s40246-015-0055-x

**Published:** 2015-12-14

**Authors:** Eileen C. P. Lim, Maggie Brett, Angeline H. M. Lai, Siew-Peng Lee, Ee-Shien Tan, Saumya S. Jamuar, Ivy S. L. Ng, Ene-Choo Tan

**Affiliations:** KK Research Centre, KK Women’s and Children’s Hospital, 100 Bukit Timah Road, Singapore, 229899 Singapore; Genetics Service, Department of Paediatrics, KK Women’s and Children’s Hospital, Singapore, 229899 Singapore; Paediatrics Academic Clinical Programme, SingHealth Duke-NUS Graduate Medical School, Singapore, 169857 Singapore

**Keywords:** Congenital disorders, Gene panel, ICCG, Mutation screening, Next-generation sequencing

## Abstract

**Background:**

Next-generation sequencing (NGS) has revolutionized genetic research and offers enormous potential for clinical application. Sequencing the exome has the advantage of casting the net wide for all known coding regions while targeted gene panel sequencing provides enhanced sequencing depths and can be designed to avoid incidental findings in adult-onset conditions. A HaloPlex panel consisting of 180 genes within commonly altered chromosomal regions is available for use on both the Ion Personal Genome Machine® (PGM^TM^) and MiSeq platforms to screen for causative mutations in these genes.

**Methods:**

We used this Haloplex ICCG panel for targeted sequencing of 15 patients with clinical presentations indicative of an abnormality in one of the 180 genes. Sequencing runs were done using the Ion 318 Chips on the Ion Torrent PGM. Variants were filtered for known polymorphisms and analysis was done to identify possible disease-causing variants before validation by Sanger sequencing. When possible, segregation of variants with phenotype in family members was performed to ascertain the pathogenicity of the variant.

**Results:**

More than 97 % of the target bases were covered at >20×. There was an average of 9.6 novel variants per patient. Pathogenic mutations were identified in five genes for six patients, with two novel variants. There were another five likely pathogenic variants, some of which were unreported novel variants.

**Conclusions:**

In a cohort of 15 patients, we were able to identify a likely genetic etiology in six patients (40 %). Another five patients had candidate variants for which further evaluation and segregation analysis are ongoing. Our results indicate that the HaloPlex ICCG panel is useful as a rapid, high-throughput and cost-effective screening tool for 170 of the 180 genes. There is low coverage for some regions in several genes which might have to be supplemented by Sanger sequencing. However, comparing the cost, ease of analysis, and shorter turnaround time, it is a good alternative to exome sequencing for patients whose features are suggestive of a genetic etiology involving one of the genes in the panel.

## Background

Congenital disorders comprise conditions present at birth or those that developed during infancy or early childhood. Presentations include structural abnormalities, neuromuscular disorders, developmental delay, and intellectual disability which collectively affect more than 10 % of children. The European Surveillance of Congenital Anomalies (EUROCAT) reported the prevalence of major congenital anomalies to be about 2.4 % of live births [1], while the Center for Disease Control and Prevention (CDC) reported 3.3 % for birth defects [2]. The prevalence of developmental disabilities is reported to be 13.9 % in the USA [3].

Less than half of these disorders have an identifiable cause such as aneuploidy, metabolic disorder, maternal infection, parental exposure to teratogenic agents, or intrapartum events. The remaining cases are thought to have a genetic etiology such as submiscroscopic chromosomal abnormalities or rare single/multiple nucleotide changes. The former can be detected by using chromosomal microarray analysis (CMA) which is now the recommended first-tier test for children with dysmorphism, multiple congenital anomalies, developmental delay/intellectual disability, and/or autism spectrum disorder [4]. Although CMA is more sensitive than conventional karyotyping, the diagnostic yield for this group of disorders is still only about 20 % in multiple studies [5–7]. Genetic causes for the rest are likely due to small deletions and insertions, balanced translocations involving gene disruptions, and point mutations which cannot be detected by commonly used CMA platforms.

With massively parallel sequencing, many regions and even the entire genome can be interrogated simultaneously to identify such mutations. Although the cost of whole genome sequencing has become progressively lower in the last few years, data analysis and interpretation remain challenging. Due to the large number of short-reads, the sequence data has to be mapped back to the reference genome and filtered through known databases to identify variants for each individual, leading to long turnaround time from clinic testing to reporting. There is also the issue of incidental findings unrelated to the indication for testing and the American College of Medical Genetics and Genomics (ACMG) have recommended the reporting of pathogenic variants for 56 genes [8]. Subsequently, the ACMG recommended that patients be given the choice of opting out of receiving such information [9]. For these reasons, many laboratories still use Sanger sequencing of single or a few genes when there are known causal genes for the suspected disorders.

Exome sequencing can partly overcome the issue of data throughput but not the possibility of incidental findings. Targeted gene panels can address both by focusing on a set of relevant candidate genes with known diagnostic yield, while providing cost-related advantage as well as easier data analysis without the need for specialized computing infrastructure and expertise. The American Society of Human Genetics (ASHG) also recommends that gene testing should be limited to single genes or targeted gene panels based on the clinical presentations of the patient [10]. Compared to Sanger sequencing of single genes, targeted gene panel sequencing has much higher throughput, but each design needs to be evaluated for coverage and sensitivity before being put to routine clinical diagnostic use.

Among several pre-designed catalog panels for pediatric congenital disorders, there is one comprising 180 genes located within chromosomal regions with a high frequency of cytogenetic abnormalities in constitutional disorders [11] according to publicly available data from the International Collaboration for Clinical Genomics (ICCG—previously known as International Standards for Cytogenomic Arrays or ISCA) [12, 13]. To assess the coverage and sensitivity of this ICCG gene panel for high-throughput next-generation sequencing in congenital disorders, we used the Ion Torrent PGM platform to perform mutation screening of 15 pediatric patients with suspected genetic disorders.

## Materials and methods

### Ethics statement

The patients were previously recruited under two separate projects (CIRB Ref: 2007/831/F and 2010/238/F). Approval to conduct this sequencing study was provided by the SingHealth Central Institutional Review Board (CIRB Ref: 2013/798/F). All the subjects were minors, and written informed consent had been obtained from the parents.

### Study samples

The 15 patients were previously recruited from the hospital’s Genetics Clinics for testing of chromosomal imbalance using human 400 K CGH arrays (Agilent Technologies Inc., Santa Clara, USA). No significant pathogenic copy number changes were identified in all 15. Inclusion criteria include developmental delay/intellectual disability and multiple congenital anomalies. Each patient had been followed up and examined by a clinical geneticist. All of them have clinical features suggestive of a disorder associated with one of the 180 genes, although the features may not have been typical or completely fulfilled the clinical criteria of a specific syndrome at the time of recruitment.

### DNA extraction

Genomic DNA was manually extracted from peripheral blood collected in EDTA tubes using the Gentra Puregene Blood Kit (Qiagen Inc., Valencia, USA) according to the manufacturer’s instructions. DNA quality and quantity were measured on a Nanodrop Spectrophotometer (Thermo Scientific, Wilmington, USA).

### Library construction, sequencing, and data analysis

Genomic DNA (225 ng gDNA) was digested with 16 different restriction enzymes at 37 °C for 30 min to create a library of gDNA restriction fragments. Both ends of the targeted fragments were selectively hybridized to biotinylated probes from the HaloPlex ICCG panel (Agilent Technologies Inc., Santa Clara, CA, USA), which resulted in direct fragment circularization. During the 16-h hybridization process, HaloPlex ION Barcodes and Ion Torrent sequencing motifs were incorporated into the targeted fragments. Circularized target DNA-HaloPlex probe hybrids containing biotin were then captured by HaloPlex Magnetic Beads on the Agencourt SPRIPlate Super magnet magnetic plate. DNA ligase was added to close the nicks in the hybrids, and freshly-prepared NaOH was used to elute the captured target libraries. The target libraries were then amplified with 18 PCR cycles and purified using AMPure XP beads. Amplicons ranging from 150 to 550 bp were then quantified using an Agilent BioAnalyzer High Sensitivity DNA Assay kit on the 2100 Bioanalyzer to validate the enrichment of the libraries. Library preparation took approximately 1½ days.

Equimolar amounts of four multiplexed bar-coded libraries were pooled and clonally amplified by emulsion PCR, using the Ion PGM Template OT2 200 Kit 9 (Life Technologies, Carlsbad, CA, USA). The template-positive Ion Sphere Particles (ISPs) were then enriched with the Ion OneTouch^TM^ ES and loaded on an Ion 318^TM^ Chip v1. Four separate runs were performed for the 15 samples, with one sample sequenced twice on two different chips. Sequencing was carried out in the Ion PGM^TM^ System using the Ion PGM^TM^ Sequencing 200 Kit v2 according to the manufacturer’s instructions with 500 flow runs.

The data from the sequencing runs were analyzed using the Torrent Suite v4.0.2 analysis pipeline, which includes raw sequencing data processing (DAT processing), splitting of the reads according to the barcode for the individual sample output sequence, classification, signal processing, base calling, read filtering, adapter trimming, and alignment QC. Single-nucleotide polymorphisms (SNP), multi-nucleotide polymorphisms (MNPs), insertions, and deletions were identified across the targeted subset of the reference using a plug-in Torrent Variant Caller (v4.0-r76860), with the parameter settings optimized for germ-line high frequency variants and minimal false positive calls. The output variant call format (VCF) file was then annotated through the web-based user-interfaced GeneTalk (GeneTalk GmbH, Berlin, Germany) and Ensembl Variant Effect Predictor [14].

Sequence variants were compared with data in dbSNP, 1000 Genomes and Human Genome Mutation Database. Variants not previously reported in healthy controls or previously classified as pathogenic were evaluated for coverage depth and also visually inspected using the Integrative Genomics Viewer before validation by dideoxy sequencing using standard protocol for BigDye® Terminator v3.1 Cycle Sequencing Kit (Life Technologies, Carlsbad, CA, USA). Segregation analysis was performed when DNA from family members was available. Sequencing was carried out on the Applied Biosystems® 3130 Genetic Analyzer (Life Technologies, Carlsbad, CA, USA). In addition, SIFT (sift.bii.a-star.edu.sg) and Polyphen2 (genetics.bwh.harvard.edu/pph2) were used to check the likely functional significance of missense variants for clinical interpretation.

## Results

**Fig. 1:**
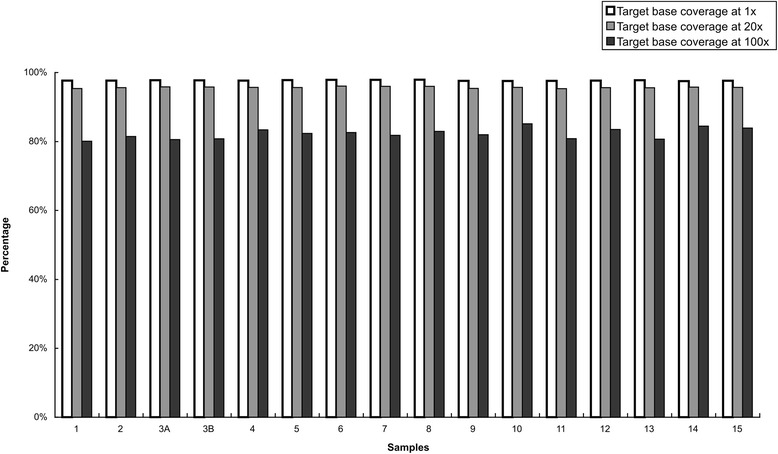
Percentage of bases at the different read depths

An average of 790 Mb was generated per chip (range 748–828 Mb). Loading densities of the targeted sequencing of four libraries (four samples were multiplexed in each library) ranged from 75 to 81 %. The total number of reads (usable sequence) ranged from 5.8 to 6.4 M, and average read length ranged from 124 to 131 bp. After filtering out polyclonal, low quality, and primer dimers, the percentage of usable reads ranged from 69 to 73 %. On average, each sample yielded 196 M bases from 1.5 M reads (Table 1 and Fig. 1) from 58,670 amplicons with a mean read length of 128 bp. One sample was sequenced twice, with near identical output obtained for both runs. The numbers of reads were 1,552,042 and 1,556,202 for total reads and 1,522,728 and 1,524,576 for mapped reads, and total numbers of bases sequenced were 199,024,281 and 200,813,003.

**Table 1:** Summary of sequencing output and quality for each sample

Sample	Reads				Bases			
	Total	Mapped	On target	Mean depth	Aligned	≥Q20	On target	Uniformity^a^
1	1,348,756	1,322,761	91.29 %	203.4	98.59 %	87.61 %	55.36 %	92.47 %
2	1,389,395	1,361,138	91.29 %	209.9	98.58 %	87.80 %	54.95 %	92.55 %
3	1,552,042	1,522,728	91.16 %	234.3	98.63 %	87.82 %	55.29 %	92.37 %
4	1,494,165	1,470,215	91.90 %	226.8	98.71 %	87.87 %	55.06 %	92.76 %
5	1,369,435	1,346,412	91.91 %	210.2	98.78 %	88.89 %	54.65 %	92.90 %
6	1,663,702	1,633,814	91.20 %	252.4	98.72 %	88.33 %	55.03 %	92.43 %
7	1,602,753	1,569,980	91.01 %	242.7	98.67 %	88.75 %	55.14 %	92.36 %
8	1,694,348	1,662,379	91.25 %	256.8	98.69 %	88.80 %	54.90 %	92.36 %
9	1,431,017	1,398,943	90.08 %	211.7	98.30 %	88.04 %	54.83 %	92.52 %
10	1,717,174	1,677,112	90.16 %	253.2	98.24 %	87.83 %	55.57 %	92.12 %
11	1,408,352	1,373,789	89.67 %	205.5	98.12 %	87.42 %	55.28 %	92.56 %
12	1,511,078	1,484,377	90.97 %	227.3	98.42 %	88.06 %	54.93 %	92.51 %
13	1,554,866	1,521,948	90.96 %	235.1	98.44 %	89.17 %	55.07 %	92.11 %
14	1,578,886	1,547,559	91.48 %	239.6	98.54 %	89.31 %	55.09 %	92.54 %
15	1,558,185	1,525,061	90.91 %	234.0	98.50 %	88.91 %	55.03 %	92.40 %

^a^Percentage of target bases covered by at least 0.2× the average base read length

Approximately 97.4 % of the reads were aligned to the reference genome (hg19) and 91 % mapped to the target regions, with average base coverage ranging from 203× to 256× for individual samples. 97.7 % of the targets had minimum read depth of 20×, 95.6 % at >50× and 88.2 % at >100×. Full coverage was achieved for more than 95 % of targets in all 15 samples, and most (approximately 89.9 %) target bases did not show any bias toward forward or reverse strand read alignment. The average total coverage of all targeted bases was 95.7 % at 20× and 82.38 % at 100×. Coverage was also uniform across all samples. More than 88 % of called bases had a quality score of ≥Q20 (Table 1).

**Fig. 2:**
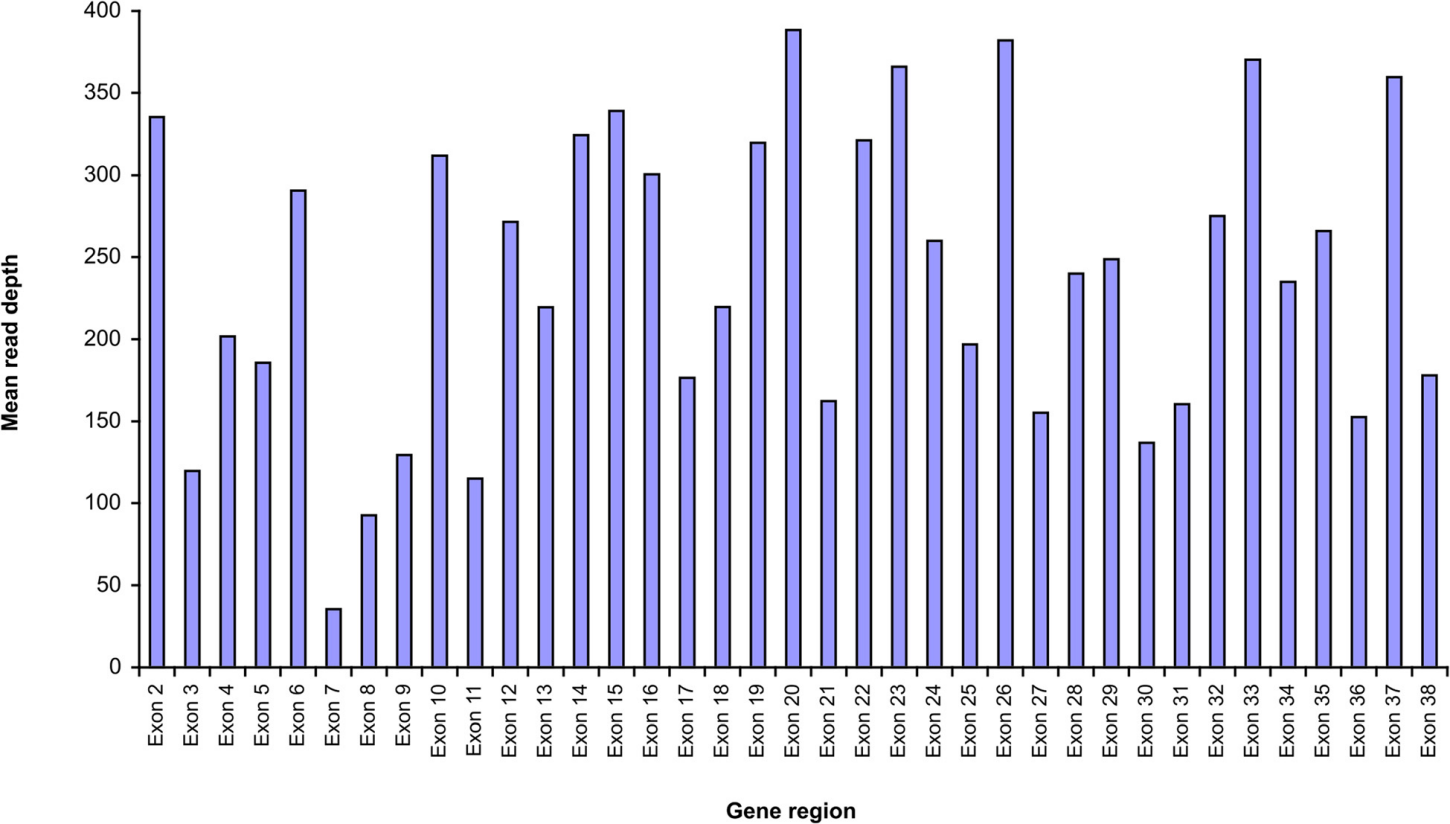
Average target base read depth for exons 2–38 of *CHD7*

**Fig. 3:**
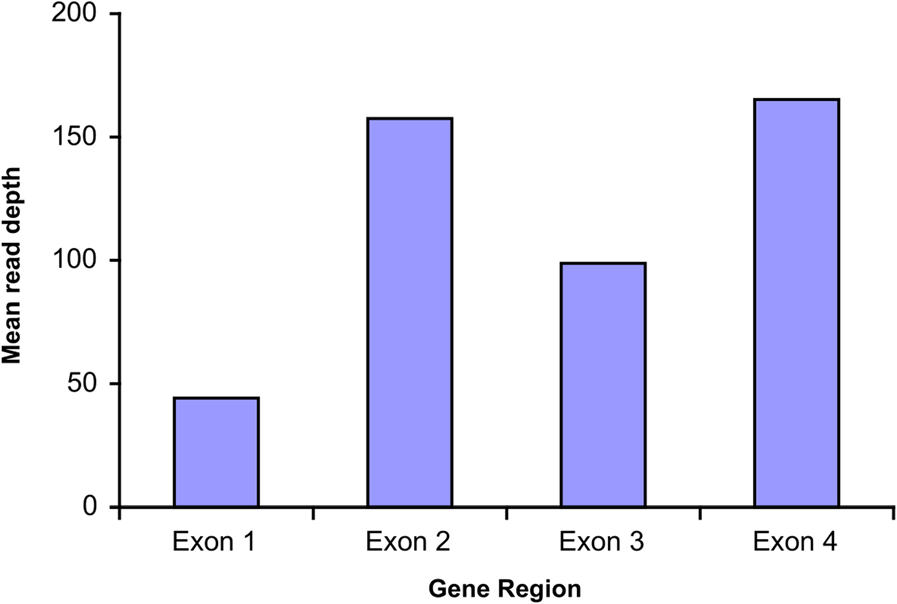
Average target base read depth for exons 1–4 of *MECP2*

**Fig. 4:**
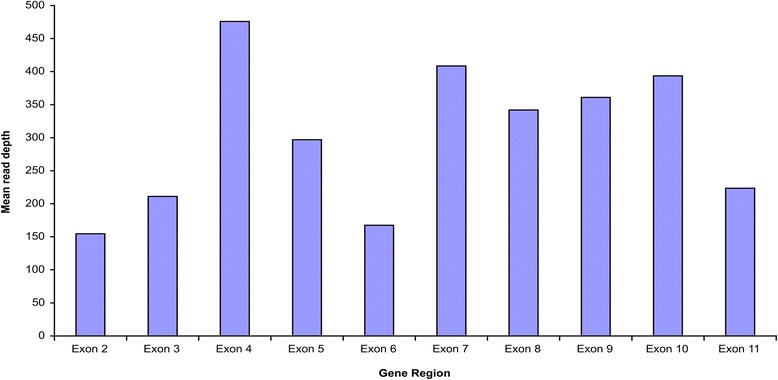
Average target base read depth for exons 2–11 of *SATB2*

At the gene level, 137 of the 180 genes had mean coverage of at least 20×, of which 99 had a mean of >50× and 40 had a mean of >100× (Table 2). Despite the high target region coverage, amplification failed for at least 26 exons across the 180 genes. Thirteen genes (*CFC1*, *CHRNA7*, *CYP21A2*, *EHMT1*, *F8*, *HBA1*, *HBA2*, *IKBKG*, *NOTCH2*, *PKD1*, *SGCE*, *SRY*, *TSC2*) had at least one region that was not amplified and therefore not sequenced (lowest number of reads “0” in Table 2). The sequencing coverage of *CFC1*, *IKBKG*, *HBA1*, and *HBA2* was low with >50 % of these genes sequenced at >20× (Table 3). The gene with the highest mean coverage was *SALL1* (358×). The poorest coverage was for *CFC1*. Mean read depth for individual exons for three different genes were shown in Figs. 2, 3, and 4.

**Table 2:** Mean coverage with highest and lowest number of reads for target regions for each gene

	Gene	Mean	Lowest	Highest
1	*ABCC8*	338.07	81.92	786.09
2	*ABCD1*	169.66	12.56	411.39
3	*ACSL4* ^a^	164.77	21.30	492.11
4	*AFF2*	214.46	36.76	580.05
5	*ALX4*	222.07	84.73	558.92
6	*AP1S2* ^a^	135.94	38.59	325.08
7	*APC* ^a^	179.90	3.73	406.62
8	*AR*	223.99	43.85	529.40
9	*ATP7A* ^a^	178.46	15.96	431.28
10	*ATRX*	158.16	10.57	441.01
11	*AVPR2*	212.24	91.24	401.30
12	*BMP4* ^a^	277.08	184.26	355.73
13	*BMPR1A* ^a^	249.32	92.33	500.82
14	*BMPR2*	221.72	39.06	545.08
15	*BRCA2* ^a^	226.44	69.62	659.97
16	*BRWD3*	158.01	1.94	403.65
17	*BSND*	281.21	166.64	426.50
18	*BTK* ^a^	248.63	71.65	522.36
19	*CACNA1C*	313.63	70.18	681.23
20	*CASK*	174.65	3.07	469.39
21	*CDKN1C* ^a^	61.17	21.98	111.66
22	*CFC1*	0.00	0.00	0.06
23	*CHD7* ^a^	238.95	6.00	491.12
24	*CHD8* ^a^	241.14	3.17	571.36
25	*CHM*	138.15	0.00	424.06
26	*CHRNA7*	133.17	0.00	649.69
27	*CLCNKA* ^a^	207.37	41.54	632.50
28	*CLCNKB* ^a^	227.42	19.10	558.00
29	*CNTN4* ^a^	258.10	74.48	742.09
30	*COL2A1*	311.23	28.83	762.32
31	*COL4A5*	145.99	6.30	492.06
32	*CREBBP*	307.73	66.01	682.75
33	*CUL4B* ^a^	148.17	35.09	399.12
34	*CYP21A2*	42.13	0.00	317.76
35	*DCX*	191.11	31.11	424.96
36	*DHCR7* ^a^	356.18	73.73	715.42
37	*DMRT1*	317.71	99.58	526.08
38	*DYM* ^a^	199.54	35.51	538.64
39	*DYRK1A*	238.22	56.46	539.50
40	*EDNRB* ^a^	244.22	108.94	440.60
41	*EHMT1*	322.86	0.00	914.42
42	*EMX2*	191.49	89.92	367.85
43	*EXT1*	255.03	122.82	531.24
44	*EXT2*	268.11	55.88	603.23
45	*EYA1*	259.46	9.39	471.54
46	*F8*	208.53	0.00	590.76
47	*F9*	194.99	29.24	362.11
48	*FANCA*	305.66	17.51	898.72
49	*FANCB* ^a^	115.90	28.51	270.95
50	*FBN1* ^a^	275.76	42.56	611.18
51	*FGD1*	232.34	56.74	586.70
52	*FGFR1* ^a^	313.99	118.45	666.21
53	*FLNA* ^a^	243.40	56.48	688.98
54	*FMR1*	156.86	48.89	329.36
55	*FOXC1*	93.65	76.19	114.03
56	*FOXG1*	96.79	77.95	125.85
57	*FOXL2*	93.73	75.81	114.31
58	*FZD4*	211.12	93.71	360.16
59	*GATA3* ^a^	324.33	155.81	637.81
60	*GATA4* ^a^	295.62	50.09	587.59
61	*GDF5*	155.05	113.49	212.75
62	*GJB2* ^a^	249.56	200.76	298.70
63	*GLA*	214.53	82.27	473.23
64	*GLI2*	312.00	149.69	577.16
65	*GLI3* ^a^	286.99	108.72	560.08
66	*GPC3*	216.44	42.47	424.44
67	*GPC6*	251.39	134.79	392.07
68	*GPR56* ^a^	294.55	71.00	606.66
69	*GRIA3*	204.51	69.61	433.75
70	*HBA1*	50.15	0.00	182.69
71	*HBA2*	10.42	0.00	40.79
72	*HCCS* ^a^	177.03	62.86	347.20
73	*HNF1B*	321.62	59.22	722.67
74	*HOXD13*	278.61	105.47	524.08
75	*HPRT1*	151.03	46.04	318.21
76	*IDS*	183.74	4.69	498.62
77	*IKBKG*	51.43	0.00	348.87
78	*IRF6* ^a^	265.75	126.04	468.45
79	*JAG1*	308.99	57.19	644.27
80	*KAL1*	196.43	33.50	435.56
81	*KCNJ1*	341.18	200.92	557.14
82	*KCNQ1*	321.89	62.66	842.39
83	*L1CAM* ^a^	238.10	33.83	531.65
84	*LAMP2*	161.71	16.59	374.76
85	*LEMD3*	162.74	55.42	325.70
86	*LHX4*	340.77	146.93	643.51
87	*LMX1B*	165.54	66.47	408.35
88	*MECP2*	116.40	21.60	224.22
89	*MID1* ^a^	188.60	44.28	383.24
90	*MITF*	303.77	97.21	559.04
91	*MSX1*	148.47	85.95	232.61
92	*MSX2*	147.01	93.77	230.81
93	*MTM1* ^a^	197.51	54.42	517.75
94	*MYCN* ^a^	228.84	96.40	407.08
95	*NDP* ^a^	237.17	92.91	444.75
96	*NDUFV1*	299.67	104.88	555.45
97	*NF2*	368.03	144.63	794.05
98	*NHS*	189.40	24.18	373.29
99	*NIPBL* ^a^	172.28	15.79	382.14
100	*NLGN4X* ^a^	251.27	119.13	470.66
101	*NOTCH2*	281.99	0.00	642.59
102	*NR5A1* ^a^	222.01	116.84	407.74
103	*NRXN1* ^a^	225.80	21.56	577.97
104	*NSD1* ^a^	261.44	86.24	500.38
105	*OCA2* ^a^	321.02	106.42	685.92
106	*OCRL*	178.97	13.16	440.97
107	*OFD1*	171.17	58.00	394.13
108	*OTC*	190.78	1.46	562.10
109	*OTX2*	311.41	198.51	476.69
110	*PAFAH1B1* ^a^	234.58	15.99	516.41
111	*PAK3*	181.03	47.33	405.32
112	*PAX3*	235.81	80.62	569.70
113	*PAX6* ^a^	242.37	25.79	599.28
114	*PAX9* ^a^	258.47	7.00	540.15
115	*PGK1*	252.23	95.17	614.74
116	*PHEX*	200.00	48.82	439.78
117	*PHF6* ^a^	165.22	63.07	315.44
118	*PIGB*	205.86	19.26	539.76
119	*PITX2* ^a^	283.28	122.96	548.91
120	*PKD1*	99.18	0.00	512.88
121	*PKD2*	225.02	47.45	475.99
122	*PLP1*	247.34	9.76	525.11
123	*PREPL* ^a^	215.09	36.12	484.89
124	*PRPS1*	247.30	102.55	418.52
125	*PTCH1*	270.35	12.72	733.37
126	*PTEN*	150.42	22.31	371.28
127	*PTPN11*	267.28	7.24	610.72
128	*RAI1* ^a^	343.75	101.17	648.66
129	*RB1*	131.02	14.38	388.44
130	*RET*	271.31	71.71	635.08
131	*RPS19* ^a^	321.73	108.91	519.38
132	*RS1*	222.95	115.25	327.74
133	*RUNX2* ^a^	273.57	111.60	506.80
134	*SALL1* ^a^	358.75	261.01	497.61
135	*SALL4*	275.10	113.08	406.79
136	*SATB2* ^a^	308.41	180.72	485.31
137	*SCN1A*	184.22	17.84	385.17
138	*SGCE*	176.87	0.23	424.34
139	*SH2D1A*	221.35	64.88	432.85
140	*SHANK3*	244.90	12.74	621.93
141	*SHH*	161.98	64.47	259.27
142	*SIX3*	122.83	85.98	168.90
143	*SLC12A1* ^a^	258.77	62.97	558.39
144	*SLC12A3*	280.89	56.97	814.03
145	*SLC16A2*	265.88	60.08	534.50
146	*SLC3A1*	226.04	90.48	481.54
147	*SLC6A8*	115.64	2.00	346.38
148	*SLC9A6*	140.59	40.48	373.83
149	*SMAD4* ^a^	290.53	109.10	607.62
150	*SOX2*	195.01	156.36	240.25
151	*SPINK1* ^a^	194.39	57.54	401.97
152	*SRY*	65.35	0.00	189.58
153	*SYN1*	211.18	33.53	488.47
154	*SYNGAP1*	209.23	41.07	495.34
155	*TBCE* ^a^	274.69	89.15	774.41
156	*TBX1* ^a^	281.13	28.78	628.34
157	*TBX3*	229.37	102.59	459.90
158	*TBX5* ^a^	267.57	94.82	473.34
159	*TCF4* ^a^	277.43	72.17	568.07
160	*TCOF1*	335.67	175.06	602.11
161	*TGFBR1*	211.80	1.52	479.34
162	*TGFBR2*	303.18	84.26	614.98
163	*TGIF1*	302.73	169.50	481.36
164	*TIMM8A*	166.52	37.46	370.74
165	*TRPS1* ^a^	267.79	112.32	411.15
166	*TSC1* ^a^	293.98	45.37	607.17
167	*TSC2* ^a^	286.10	0.00	776.60
168	*TWIST1*	96.89	76.02	120.38
169	*UPF3B*	200.15	53.63	424.96
170	*USH1C*	283.03	38.50	767.39
171	*VHL*	130.75	57.24	269.80
172	*WT1*	335.43	112.21	654.89
173	*XIAP* ^a^	132.08	31.85	278.12
174	*ZDHHC9* ^a^	223.57	61.28	554.63
175	*ZEB2* ^a^	261.68	97.71	474.05
176	*ZFPM2*	211.09	6.00	393.25
177	*ZIC1*	228.52	127.92	369.74
178	*ZIC2*	128.92	29.81	320.70
179	*ZIC3*	202.01	120.08	320.90
180	*ZIC4*	291.25	127.92	616.00

^a^Target regions do not include non-coding first exons

**Table 3:** Percentage of coverage for each gene at 20×

*ABCC8*	100.00 %	*DMRT1*	100.00 %	*HNF1B*	100.00 %	*OTX2*	100.00 %	*SLC16A2*	100.00 %
*ABCD1*	100.00 %	*DYM*	100.00 %	*HOXD13*	100.00 %	*PAFAH1B1*	100.00 %	*SLC3A1*	100.00 %
*ACSL4*	100.00 %	*DYRK1A*	100.00 %	*HPRT1*	100.00 %	*PAK3*	100.00 %	*SLC6A8*	94.71 %
*AFF2*	100.00 %	*EDNRB*	100.00 %	*IDS*	89.40 %	*PAX3*	100.00 %	*SLC9A6*	100.00 %
*ALX4*	100.00 %	*EHMT1*	99.47 %	*IKBKG*	26.71 %	*PAX6*	100.00 %	*SMAD4*	100.00 %
*AP1S2*	100.00 %	*EMX2*	100.00 %	*IRF6*	100.00 %	*PAX9*	99.61 %	*SOX2*	100.00 %
*APC*	98.72 %	*EXT1*	100.00 %	*JAG1*	100.00 %	*PGK1*	100.00 %	*SPINK1*	100.00 %
*AR*	100.00 %	*EXT2*	100.00 %	*KAL1*	100.00 %	*PHEX*	100.00 %	*SRY*	100.00 %
*ATP7A*	100.00 %	*EYA1*	100.00 %	*KCNJ1*	100.00 %	*PHF6*	100.00 %	*SYN1*	100.00 %
*ATRX*	99.29 %	*F8*	99.66 %	*KCNQ1*	100.00 %	*PIGB*	100.00 %	*SYNGAP1*	100.00 %
*AVPR2*	100.00 %	*F9*	100.00 %	*L1CAM*	100.00 %	*PITX2*	100.00 %	*TBCE*	100.00 %
*BMP4*	100.00 %	*FANCA*	100.00 %	*LAMP2*	100.00 %	*PKD1*	86.06 %	*TBX1*	100.00 %
*BMPR1A*	100.00 %	*FANCB*	100.00 %	*LEMD3*	100.00 %	*PKD2*	100.00 %	*TBX3*	100.00 %
*BMPR2*	100.00 %	*FBN1*	100.00 %	*LHX4*	100.00 %	*PLP1*	79.74 %	*TBX5*	100.00 %
*BRCA2*	100.00 %	*FGD1*	100.00 %	*LMX1B*	100.00 %	*PREPL*	100.00 %	*TCF4*	100.00 %
*BRWD3*	99.43 %	*FGFR1*	100.00 %	*MECP2*	100.00 %	*PRPS1*	100.00 %	*TCOF1*	100.00 %
*BSND*	100.00 %	*FLNA*	100.00 %	*MID1*	100.00 %	*PTCH1*	97.80 %	*TGFBR1*	93.58 %
*BTK*	100.00 %	*FMR1*	100.00 %	*MITF*	100.00 %	*PTEN*	100.00 %	*TGFBR2*	100.00 %
*CACNA1C*	100.00 %	*FOXC1*	100.00 %	*MSX1*	100.00 %	*PTPN11*	89.17 %	*TGIF1*	100.00 %
*CASK*	94.17 %	*FOXG1*	100.00 %	*MSX2*	100.00 %	*RAI1*	100.00 %	*TIMM8A*	100.00 %
*CDKN1C*	100.00 %	*FOXL2*	100.00 %	*MTM1*	100.00 %	*RB1*	100.00 %	*TRPS1*	100.00 %
*CFC1*	0.00 %	*FZD4*	100.00 %	*MYCN*	100.00 %	*RET*	100.00 %	*TSC1*	100.00 %
*CHD7*	100.00 %	*GATA3*	100.00 %	*NDP*	100.00 %	*RPS19*	100.00 %	*TSC2*	98.30 %
*CHD8*	99.11 %	*GATA4*	100.00 %	*NDUFV1*	100.00 %	*RS1*	100.00 %	*TWIST1*	100.00 %
*CHM*	95.10 %	*GDF5*	100.00 %	*NF2*	100.00 %	*RUNX2*	100.00 %	*UPF3B*	100.00 %
*CHRNA7*	84.46 %	*GJB2*	100.00 %	*NHS*	100.00 %	*SALL1*	100.00 %	*USH1C*	100.00 %
*CLCNKA*	100.00 %	*GLA*	100.00 %	*NIPBL*	100.00 %	*SALL4*	100.00 %	*VHL*	100.00 %
*CLCNKB*	100.00 %	*GLI2*	100.00 %	*NLGN4X*	100.00 %	*SATB2*	100.00 %	*WT1*	100.00 %
*CNTN4*	100.00 %	*GLI3*	100.00 %	*NOTCH2*	95.39 %	*SCN1A*	100.00 %	*XIAP*	100.00 %
*COL2A1*	100.00 %	*GPC3*	100.00 %	*NR5A1*	100.00 %	*SGCE*	94.86 %	*ZDHHC9*	100.00 %
*COL4A5*	98.76 %	*GPC6*	100.00 %	*NRXN1*	100.00 %	*SH2D1A*	100.00 %	*ZEB2*	100.00 %
*CREBBP*	100.00 %	*GPR56*	100.00 %	*NSD1*	100.00 %	*SHANK3*	96.32 %	*ZFPM2*	98.84 %
*CUL4B*	100.00 %	*GRIA3*	100.00 %	*OCA2*	100.00 %	*SHH*	100.00 %	*ZIC1*	100.00 %
*CYP21A2*	67.67 %	*HBA1*	30.07 %	*OCRL*	100.00 %	*SIX3*	100.00 %	*ZIC2*	100.00 %
*DCX*	100.00 %	*HBA2*	30.07 %	*OFD1*	100.00 %	*SLC12A1*	100.00 %	*ZIC3*	100.00 %
*DHCR7*	100.00 %	*HCCS*	100.00 %	*OTC*	91.74 %	*SLC12A3*	100.00 %	*ZIC4*	100.00 %

Overall, 2326 single-nucleotide variants (SNVs) and 25 indels were identified in the 15 patients. These variants identified from the Ion Reporter had an average coverage of 595× and an average Qscore of 38. Variant annotation indicated that 2203 were common variants present in dbSNP and 1000 Genome Project databases. The number of variants ranged from 154 to 175 per patient, with an average of 9.6 novel variants each. Synonymous variants were the most common.

Variants were prioritized for Sanger confirmation based on the individual’s clinical presentations. Pathogenic variants were confirmed in six patients. The identified *CHD7* (two patients), *SHH*, *TCF4*, *TSC2*, and *MECP2* variants and the clinical features of these six patients are listed in Table 4. Another five patients had candidate variants for which further evaluation and segregation analysis are ongoing.

**Table 4:** Pathogenic variants identified and the respective patients’ associated clinical features

Patient	Gender	Age^a^	Gene	Nucleotide change	Amino acid change	Clinical features
1	M	1d	CHD7	NM_017780.3:c.7891C > T	p.R2631X	Hypoplastic left heart, choanal atresia, oesophageal atresia
2	F	1y4m	CHD7	NM_017780.3c.601C > T	p.Q201X	PDA, aortic stenosis, coloboma, hypotonia
3	F	3y9m	MECP2	NM_004992.3:c.763C > T	p.R255X	Developmental delay, hypotonia, neurodevelopmental regression, epilepsy
4	F	2w	SHH	NM_000193.3:c.413C > A	p.S138Y	Alobar HPE, PDA, hypotelorism, single nostril, choanal atresia, overlapping fingers
5	M	5y11m	TCF4	NM_001083962.1:c.1739G > A	p.R580Q	GDD, microcephaly, epicanthic folds, hypertelorism, drooling, no speech
6	F	5y8m	TSC2	NM_000548.3:c.3364delC	p.R1121Vfs*69	Bilateral large renal cysts, ballotable left kidney, cardiac rhabdomyoma, iris pigmentation & hamartomas, epilepsy

*GDD* global developmental delay, *HPE* holoprosencephaly, *PDA* patent ductus arterio

^a^Age at enrollment (d = day, y = year, m = month)

## Discussion

The HaloPlex ICCG panel is a pre-designed made-to-order panel targeting 180 genes. It follows the ICCG recommendations for design and resolution and is available through SureDesign from Agilent Technologies. The targeted panel includes genes in the most commonly altered chromosomal regions according to the ISCA/ICCG database. The 180 genes are covered by 2509 target regions which range in size from 2 to 6575 nucleotides. Depending on its size, a region is covered by between 1 and 547 amplicons.

The recommended minimum read depth for clinical diagnostic sequencing is 20× [15, 16], which was achieved for over 90 % of the target for 170 genes. For *CHD7*, even the exon with the poorest coverage had a mean of 36 (Fig. 2). Of the remaining ten, four genes had 80–90 % coverage, and the other six (*CFC1*, *CYP21A*, *HBA1*, *HBA2*, *IKBKG*, *NOTCH2*, *PLP1*) had <80 %. More than half of the targets in these individual genes are within GC-rich regions. Less efficient PCR for these templates might have resulted in sequencing failure during library preparation, or insufficient sequence data were produced [17]. In addition, the HaloPlex protocol uses restriction enzymes which are sequence-dependent and nonrandom, this method might have contributed further to uneven coverage and also gaps in coverage [18]. For *IKBKG*, the presence of a pseudogene might have caused non-specific alignment and contributed to the low capture of target sequences [19]. Nijman et al. have almost no mapped reads in *IKBKG* in their targeted sequencing, and generally poor coverage of *CFC1* and *IKBKG* had been reported in multiple studies [20–22]. For the gene with the poorest coverage *CFC1*, all six exons had no reads across all 15 samples. This gene is associated with the generation of left-right asymmetry via the TGF pathway. There were 23 mutations in HGMD, 13 of which were found in patients with congenital heart disease [23]. This panel would not be useful for patients with clinical suspicion of *CFC1* gene mutations.

The first exon of 64 genes was not included in the design (indicated with “*” in Table 2). All the 64 genes have one or more non-coding exon. The entire exon 1 of these genes (and additional exons for some others) contains only untranslated regions. In general, amplification of exon 1 of some genes was problematic because of the generally higher GC content and sequence complexity [24–26]. Our results showed that *MECP2* had an average target base read depth of 118×. The coverage for exon 1 is the lowest among all, but it is still two times that of the minimum of 20× recommended for clinical diagnostics (Fig. 3). *SATB2* had an average target base read depth of 300×, but exon 1 was not covered in the design (Fig. 4). Nevertheless, including non-coding exons in the design might improve the yield of NGS as variants affecting splicing of non-coding exons have been reported to be disease-causing [27].

Many congenital disorders do not have unique and exclusive features, and the presentations may be non-specific. Even for syndromic disorders, there are overlapping features, and the phenotypic features in some patients may be atypical, making it challenging for the clinical geneticists to come to a diagnosis based on clinical history and examination. All the 15 patients in this study have constitutional disorders and suspicion of chromosomal disorders, but CMA did not find any pathogenic copy number abnormality. With this targeted panel, we were able to reach a molecular diagnosis for six patients after reviewing the results with their primary physicians (Table 4). Pathogenic *CHD7* variants were detected in two patients with clinical features consistent with CHARGE syndrome. Both *CHD7* variants identified (p.R2613X and p.Q201X) have been previously reported in other CHARGE patients [28]. A pathogenic p.R255X *MECP2* variant was detected in a patient with clinical features of Rett syndrome. This variant has also been reported previously [29]. The patients with the truncating *TSC2* variant and the missense *SHH* variant also showed clinical features consistent with the respective causative genes. These two variants are novel and the missense variant is predicted to be pathogenic according to both SIFT and Polyphen. Similarly, the clinical features of the patient with the *TCF4* variant are found to be consistent with Pitt-Hopkins syndrome upon retrospective review of the patient’s progressive features by the attending physician. This p.R580Q *TCF4* variant has been reported as pathogenic in patients with Pitt-Hopkins syndrome [30].

The identification of a patient’s causative mutation has the translational benefit of providing the parents with an answer for their child’s condition. In addition, it provides a guide to the attending clinician on the management and prognosis of the patient. A molecular diagnosis would also facilitate access to clinical trials and programs for special needs children. The use of appropriate gene panels obviates the need for subjective clinical decision on which gene(s) to test in each patient, and may lead to a standard testing workflow for each group of disorders. Generally for those whose diagnosis can be narrowed down to a few suspected genetic syndromes, targeted gene panels would be superior to exome sequencing which has more limitations in the diagnostic setting due to coverage deficiencies in some genes and longer turnaround time. Higher-average read depth could be attained at a lower cost, making it superior to exome sequencing in terms of cost, sensitivity, and expected diagnostic yield [31, 32].

## Conclusions

The Haloplex ICCG panel had good coverage except for ten of the target genes. Consideration would have to be made for the low coverage for some regions in several genes which might have to be supplemented by Sanger sequencing. However, comparing the cost, ease of analysis, and shorter turnaround time, it is a good alternative to exome sequencing for patients whose features are suggestive of a genetic etiology involving one of the genes in the panel.
